# Lectin-Based Method for Deciphering Human Milk IgG Sialylation

**DOI:** 10.3390/molecules24203797

**Published:** 2019-10-22

**Authors:** Jolanta Lis-Kuberka, Barbara Królak-Olejnik, Marta Berghausen-Mazur, Magdalena Orczyk-Pawiłowicz

**Affiliations:** 1 Department of Chemistry and Immunochemistry, Wroclaw Medical University, M. Skłodowskiej-Curie 48/50, 50-369 Wrocław, Poland; jolanta.lis-kuberka@umed.wroc.pl; 2 Department and Clinic of Neonatology, Wroclaw Medical University, Borowska 213, 50-556 Wrocław, Poland; barbara.krolak-olejnik@umed.wroc.pl; 3 Department of Pediatrics, Division of Neonatology, Wroclaw Medical University, Bartla 5, 51-618 Wroclaw, Poland; Marbemaz@gmail.com

**Keywords:** lectins, molecular probes, glycoconjugates recognition, glycocompounds, biomedical and biopharmaceutical applications, human lactation, human milk, immunoglobulin G, sialylation

## Abstract

In light of the immunoprotective function of human milk and the incontestable impact of IgG glycosylation on its immune functions, characterization of the sialylation profile of human milk IgG is needed. Lectins as a molecular probe were applied in lectin-IgG-ELISA to analyze the sialylation and galactosylation pattern of skim milk IgG of mothers who delivered at term and prematurely. Well-defined biotinylated lectins were used: *Maackia amurensis* II (MAA II), *Sambucus nigra* (SNA), *Ricinus communis* I (RCA I), and *Griffonia simplicifolia* II (GSL II) specific to α2,3-Neu5Ac, α2,6-Neu5Ac, Gal(β1,4)GlcNAc, and agalactosylated glycans, respectively. The sialylation pattern of milk IgG differs qualitatively and quantitatively from maternal plasma IgG and is related to lactation stage and perinatal risk factors. Expression of MAA-, SNA-, and GSL-reactive glycotopes on term milk IgG showed a positive correlation with milk maturation from days 1 to 55. Preterm birth was associated with an increase of MAA-reactive and a decrease of RCA-reactive IgG glycotopes. Moreover, higher SNA- and GSL-reactive and lower RCA-reactive glycoform levels of milk IgG were associated with infection of lactating mothers. Application of a specific and simple method, lectin-IgG-ELISA, reveals the sialylation pattern of milk IgG over milk maturation. However, further investigations are needed in this area.

## 1. Introduction

Thanks to the use of lectins in studies of glycoproteins [1,2,3], knowledge concerning the glycosylation pattern of milk glycoproteins is constantly expanding [4,5,6,7,8]. The lectin-based ELISA approach has been used with very high efficiency in the characterization of the glycosylation profile for over 25 years and allows bypassing cost-intensive and time-consuming procedures associated with the isolation of glycoproteins. It should be pointed out that the application of such a methodology allows one to avoid the situation when the concentration of a biomolecule in a biological sample is too low for isolation and to perform analyses using techniques such as nuclear magnetic resonance and mass spectrometry [1,3,7,8]. Moreover, in contrast to the above-mentioned methods, lectins with well-defined specificity are an invaluable tool for the determination of monosaccharides terminally bonded by different linkages such as fucose and sialic acid, which are crucial for the biological function of many immunologically important glycoproteins including immunoglobulin G [9,10,11,12,13].

Human breast milk is the best nutrient recommended by the WHO for term and high-risk group preterm newborns [14,15] to ensure the proper growth, development, and maturation of digestive, nervous, and immune systems. Among the wide range of components of human milk, milk glycoproteins including immunoglobulins delivered to immunologically immature newborns during breastfeeding are involved in protection against viral and bacterial infections [9,16].

In human milk, immunoglobulin G content does not exceed 1% of total proteins, in contrast to serum, where it predominates [8,17]. Serum IgG elicits a wide range of functions [17]; however, the attached glycans are a key player in their modulation [1,18,19]. The *N*-linked glycan situated at position Asn297 in Fc of IgG is an evolutionarily conserved complex-type biantennary oligosaccharide chain, whose pentasaccharide core is galactosylated (67%) and can be decorated by the addition of core fucose (94%) and small amounts of sialic acid (18%) and bisecting GlcNAc (10%) [17,20,21,22,23]. Additionally, the *N*-glycan of the Fc fragment, human plasma IgG, can have glycosylation sites located at the antigen-binding fragment (Fab) (25% of human plasma IgG contains *N*-glycans attached to the Fab [20,22,23,24]. Detailed analysis of Fc and Fab glycans of human plasma IgG showed that their structures are different [22,23,24]. In comparison to *N*-glycans of the IgG Fc region, IgG Fab *N*-glycans have a higher content of bisecting GlcNAc (45%), galactose (94%), and sialic acid (40%) and a lower content of core fucose (69%) [22,23]. However, the presence of fucose and sialic acid has a greater impact on IgG function. Sialic acid on IgG glycans reduces affinity for Fcγ receptor III (FcγRIII, low-affinity receptor for the Fc portion of immunoglobulin G), bestowing it with anti-inflammatory properties [23,25,26]. Moreover, the amount of IgG-linked sialic acid may play an important role in converting the pro-inflammatory function of IgG into that of anti-inflammatory [25,27,28]. The latest study of Li and coworkers [29] showed that sialylation of IgG Fc *N*-glycans decreased antibody-dependent cell-mediated cytotoxicity in the context of core fucosylation, but not in its absence. In an animal model, a sialylated fraction of IgG is crucial for the anti-inflammatory properties of intravenous immunoglobulin (IVIg) [30]. The level of sialylated IgGs is decreased in the setting of autoimmunity, which promotes a pro-inflammatory state [26,27]. Interactions between sialylated glycotopes and sialic acid-specific receptors/lectins participate in modulating the function of the immune system [23,25,27,29,31]. The sialylated glycans of both Fc and Fab parts may be recognized by C-type lectins such as DC-SIGN and DCIR as well as siglecs (sialic acid-binding immunoglobulin-type lectins) such as CD22 [32,33,34].

Mother’s milk immunoglobulins transferred to newborns and infants during breastfeeding protect through specific recognition of bacterial and/or viral antigens by the Fab fragment. As reported by Sedykh and coworkers [35], the Fab fragment of milk IgGs can have two different antigen-binding sites, resulting in conformational changes to accommodate the different antigens. Moreover, similarly to IgA *N*- and *O*-glycans [9], milk IgG glycans “rinsing” epithelial cells of the throat, esophagus, and intestines of the breastfed newborn can be recognized and bound by lectin receptors of bacteria, and, as suggested by Royle et al. [9], constitute a link between the innate and adaptive immune systems.

In the light of the immunoprotective value of human milk and the incontestable impact of IgG glycosylation on its immune functions [32,34] it is important to determine the sialylation profile of human milk IgG, since it has not been analyzed previously. Recently, using lectin-IgG-ELISA, we demonstrated that the fucosylation pattern of milk IgG differs qualitatively and quantitatively from maternal plasma IgG and is related to lactation stage and perinatal risk factors [8].

The sialylated glycotopes and the presence of terminal galactose and *N*-acetylglucosamine on milk IgG glycans were analyzed in relation to lactation stages from the first/second day to the 55th/40th day by lectin-IgG-ELISA using *Maackia amurensis* (MAA), *Sambucus nigra* (SNA), *Ricinus communis* I (RCA I), and *Griffonia simplicifolia* II (GSL II) agglutinins (Vector Laboratories Inc., Burlingame, CA, USA), respectively (Table 1).

The use of lectins in lectin-IgG-ELISA was especially helpful in obtaining information about the expression of biologically active glycotopes in their conformational native form, which in vivo might be exposed and recognized by selectins of epithelial cells of newborns/infants and/or bacterial/viral lectins [3]. Moreover, such a technique has advantages, especially in monitoring the glycosylation pattern of low abundance milk glycoproteins, since a very small amount of sample is needed for analysis, which is crucial due to the limited volume of early colostrum and colostrum provided by mothers who delivered preterm.

## 2. Materials and Methods

### 2.1. Milk and Plasma Samples

The milk samples marked as “term milk” (*n* = 186) were collected from healthy lactating mothers (21–35 years old) from the first to the 55th day of lactation, who delivered healthy newborns at term (from 37 weeks, one day to 41 weeks, six days of gestation) at the 1st Department and Clinic of Gynecology and Obstetrics at Wroclaw Medical University (Wroclaw, Poland). The milk samples marked as “preterm milk” (*n* = 105) were collected from mothers (25–38 years old) from the second to 40th day of lactation, whose preterm newborn (from 26 weeks, one day to 35 weeks, six days of gestation) were hospitalized at the Department and Clinic of Neonatology at Wroclaw Medical University (Wroclaw, Poland). The exclusion criteria were as follows: using tobacco products and abusing alcohol and/or drugs. All lactating women provided their written informed consent, according to the standards accepted by the Ethics Committee at Wroclaw Medical University (KB-30/2013 and KB-411/2015).

The term milk group consisted of mothers who delivered a single healthy newborn (3360 ± 349) g, without congenital defects and genetic disorders or any symptoms of infection. A total of 52% of term pregnancies were ended by caesarean section.

The preterm milk group comprised mothers who delivered a single newborn assigned [41] to the groups:

- Extremely preterm (EP): less than 28 weeks of gestation, *n* = 5),
- Very preterm (VP): from 28 weeks, one day to 32 weeks of gestation, *n* = 40),
- Moderate preterm (MP): from 32 weeks, one day to 36 weeks of gestation, *n* = 60).

In the preterm milk group, 47% of mothers suffered from infections, 42% had a premature rupture of membranes before parturition, and 16% had hypertension during pregnancy.

Plasma samples were obtained from lactating women (*n* = 40) on the second day after delivery.

### 2.2. Sample Collection and Preparation

Mother’s milk samples of term and preterm groups were collected from the breast by manual expression and/or a breast pump once per day, in the early morning. The volume of collected milk differed significantly among mothers from 1 mL for the first to third day to almost 100 mL for the 30th–55th day of lactation. All milk samples were frozen immediately at −20 °C. The aqueous phase of milk without fat and cells, namely skim milk, was obtained by centrifugation at 3500× *g* at 4 °C for 35 minutes. The samples of skim milk and plasma were stored at −20 °C until analysis.

From the collected milk samples, based on milk maturation stage, the following groups were formed:

- early colostrum (1–3 days of lactation; *n* = 39 for term and *n* = 7 for preterm milk),
- colostrum (4–7 days of lactation; *n* = 75 for term and *n* = 15 for preterm milk),
- transitional milk (8–14 days of lactation; *n* = 43 for term and *n* = 28 for preterm milk),
- mature milk (15–55/40 days of lactation; *n* = 29 for term and *n* = 55 for preterm milk).

Moreover, milk samples of mothers who delivered preterm, due to the presence of upper respiratory tract and/or urinary infection, were classified as milk samples with infection (*n* = 50) and milk samples without infection (*n* = 55).

### 2.3. Determination of IgG Concentration

The concentration of IgG was determined using ELISA according to the procedure described previously [8]. Goat anti-human IgG antibodies (Jackson ImmunoResearch, Europe Ltd., Ely, UK) and serum IgG standard from 0.2–12.5 ng per 100 µL (Jackson ImmunoResearch) were used for analysis.

### 2.4. Lectin-IgG-ELISA

Sialic acid/galactose expression on IgG was determined by a slightly modified lectin-IgG-ELISA [42] using specific biotinylated lectins (Vector Laboratories Inc., Burlingame, USA): *Maackia amurensis* II lectin (MAA II), *Sambucus nigra* lectin (SNA), *Ricinus communis* I lectin (RCA I), and *Griffonia simplicifolia* II lectin (GSL II) showing binding preferences to α-(2,3)-Neu5Ac, α-(2,6)-Neu5Ac, Galβ-(1,4)-GlcNAc, and GlcNAc (agalactosylated) glycans, respectively (Table 1).

To quantitatively compare the reactivity of applied lectins with milk IgG glycotopes among the samples, a constant amount of IgG, namely 200 ng, based on the preliminary experiments, was used for analysis. The specificity of used lectins is not absolute and can be broader than just the terminal carbohydrate structures; however, lectin-based tests are a useful method for screening fucosylation and sialylation patterns as well as their possible changes under physiopathological conditions and during laboratory processing of glycoproteins such as IgG for therapeutic purposes [1,3,42,43].

In detail: Goat anti-human IgG antibody (Fab_2_ fragment) (Jackson ImmunoResearch) in 10 mM TBS (TRIS-buffered saline), pH 8.5 diluted 1:1000 was added to the wells of an ELISA plate (Nunc International, Naperville, IL, USA) to specifically bind and isolate IgG from a sample.

The conditions for lectin-IgG-ELISA, namely the amount of IgG and the concentration of lectins and ExtrAvidin, were confirmed in preliminary tests. For analysis, milk and plasma samples were diluted in 10 mM TBS with 10 mM CaCl_2_, 10 mM MgCl_2_, 0.05% Tween 20, and 0.5% glycerin, pH 7.5, to a final IgG concentration 2 mg/L (200 ng per well). All biological samples were assayed in duplicate. The presence of α2,3-, α2,6-sialylated, galactosylated, and agalactosylated glycotopes in IgG was analyzed with well-characterized biotin-labeled lectins MAA II (1:5000), SNA (1:5000), RCA I (1:20,000), and GSL II (1:250), respectively. The relative amounts of IgG glycotope-lectin complexes were detected by adding ExtrAvidin phosphatase-labeled (1:20,000) (Sigma, St. Louis, MO, USA), and in the next step assayed with 4-nitrophenyl phosphate (for IgG-GSL lectin the dilution of ExtrAvidin was 1:5000). The enzymatic reaction was stopped by adding 1 M NaOH and then the absorbance (AU, absorbance unit), proportional to the relative amounts of the analyzed IgG glycotope, was measured (Stat Fax 2100 Microplate Reader, Awareness Technology Inc., Palm, FL, USA) using a 405 nm filter and 630 nm as the reference filter.

In preliminary experiments, the lack of reactivity with sialic acid and galactose/agalactose specific lectins and antibody used were confirmed. The absorbances ranged from 0.03 to 0.06 depending on the lectin used. Required controls [42,44] were performed to confirmed lectins’ specificity. The coefficients of variation were calculated for lectin-IgG-ELISA, namely 5.3% and 6.1% for the intra- and inter-assay, respectively.

### 2.5. Statistical Analysis

The statistical analysis was performed with TIBCO STATISTICA 13.3 (StatSoft, Inc., Tulsa, OK, USA). Due to the higher interindividual differences reported for many factors of human milk as well as the unequal sample size in the analyzed groups, nonparametric tests were used for analysis. The results are shown as the mean ± SD and the median with 25th–75th percentiles. The Kruskal–Wallis test was used for statistical significance. The correlations were estimated according to Spearman. A two-tailed *p*-value of less than 0.05 was considered significant.

## 3. Results

### 3.1. MAA-Reactive Glycoform of Milk IgG 

**Figure 1:**
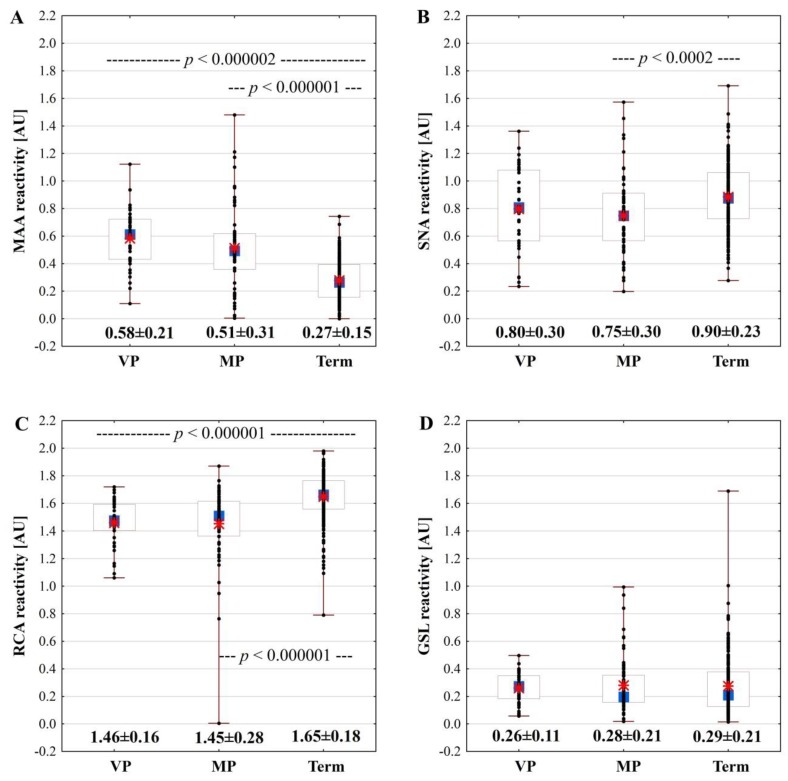
Relative amounts (AU) of *Maackia amurensis,* MAA (**A**), *Sambucus nigra* SNA (**B**), *Ricinus communis* RCA (**C**), *Griffonia simplicifolia* GSL, (**D**) reactive IgG glycoform in skim term and preterm milk. Lectin reactivity with skim milk IgG was determined as described in the Materials and Methods. VP: very preterm, and MP: moderate preterm milk; AU: absorbance units. Median (■), 25%–75% (**□**), Min–Max (I), Sample (●), Mean (❇).

The mean value of relative MAA reactivity with IgG of the term milk samples (Figure 1A) ((0.27 ± 0.15) AU) was significantly lower than the values for the very ((0.58 ± 0.21) AU; *p* < 0.000002) and moderate ((0.51 ± 0.31) AU; *p* < 0.000001) preterm milk groups (Figure 1A).

During the progression of lactation, the reactivity of MAA with IgG significantly increased from 0.16 ± 0.12 AU and 0.09 ± 0.08 AU for the early colostrum group of term and preterm milk to 0.27 ± 0.15 AU (*p* < 0.004) and 0.41 ± 0.35 AU (*p* < 0.005) in the colostrum group of the term and preterm milk, respectively. In subsequent stages of milk maturation, the reactivity with term transitional milk IgG ((0.28 ± 0.13) AU) remained almost at an unchanged level, while in the preterm group, a further increase was observed ((0.63 ± 0.24) AU; *p* < 0.003). The reactivity of MAA with term mature milk IgG was significantly higher ((0.38 ± 0.13) AU; *p* < 0.004), while in the group of preterm milk, it was significantly lower ((0.58 ± 0.22) AU; *p*< 0.00002) than in the transitional milk groups (Table 2). In contrast to milk IgG, 200 ng of lactating mothers’ plasma IgG was not recognized by MAA (Table 2).

**Figure 2:**
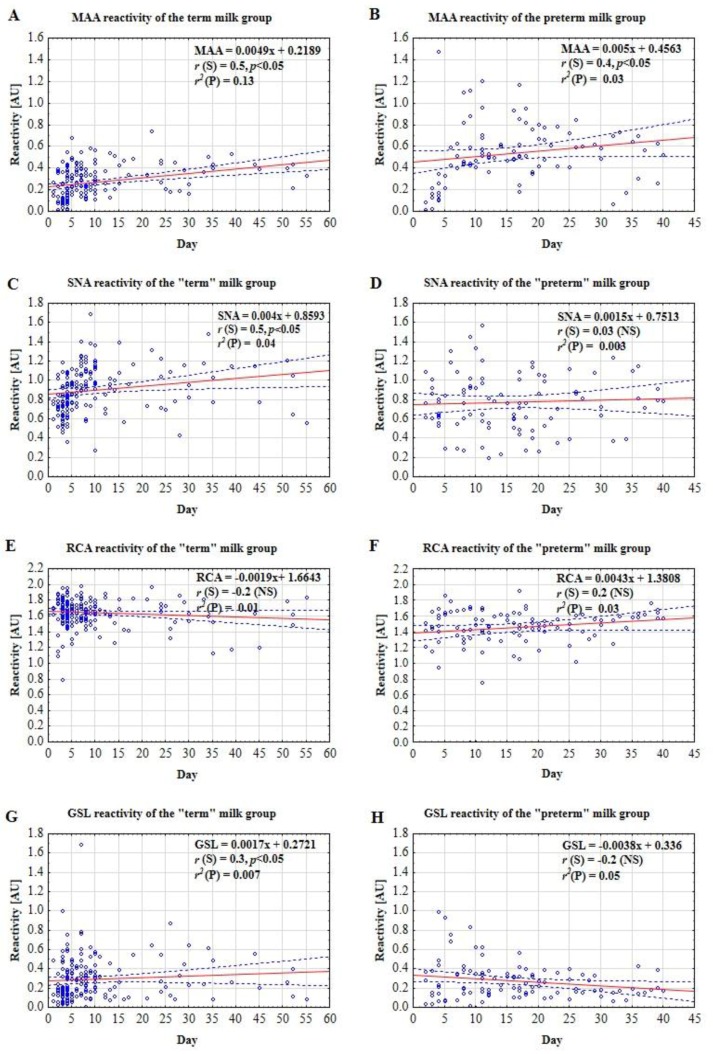
Reactivity of lectins: MAA (**A**,**B**), SNA (**C**,**D**), RCA (**E**,**F**), and GSL (**G**,**H**) with milk IgG over lactation of mothers giving birth to term and preterm newborns. A solid line indicates linear regression, and 95% confidence intervals are shown by dotted lines. The correlation coefficient (*r*) was calculated with lactation days according to Spearman and a *p*-value lower than 0.05 was regarded as significant. R-squared value is the square of the correlation coefficient of the linear regression between the day of lactation and lectin reactivity [AU]. For explanation see under Figure 1. For experimental details see under Figure 1.

The expression of MAA-reactive glycotopes on milk IgG showed a positive correlation with lactation from the 1st to 55th/40th days in both term (*r* = 0.50; *p* < 0.05) and preterm (*r* = 0.40; *p* < 0.05) groups (Figure 2A,B).

### 3.2. SNA-Reactive Glycoform of Milk IgG

The relative reactivity of SNA with milk IgG (Figure 1B) of mothers having a newborn at term ((0.90 ± 0.23) AU) was the highest and significantly different in comparison to moderate preterm ((0.75 ± 0.30) AU; *p* < 0.0002), but not with very preterm ((0.80 ± 0.30) AU) milk groups (Figure 1B).

The relative reactivity of SNA with milk IgG was 0.75 ± 0.17 AU and 0.84 ± 0.19 AU in the early colostrum of term and preterm groups, respectively. In the group of term colostrum, the mean value of relative SNA reactivity with IgG increased significantly ((0.89 ± 0.20) AU; *p* < 0005), while in the group of preterm colostrum it decreased ((0.73 ± 0.30) AU). In further milk maturation stages, the SNA reactivity with IgG of the term milk samples increased for the transitional milk group ((1.00 ± 0.25) AU, *p* < 0.006) and remained at an almost unchanged level in the group of mature milk ((0.97 ± 0.27) AU). The relative reactivity of SNA with preterm milk IgG was higher, but not significantly in the group of transitional milk ((0.81 ± 0.36) AU), while for mature milk, a subsequent drop was observed ((0.76 ± 0.28) AU) (Table 2).

The expression of SNA-reactive glycotopes on milk IgG showed a positive correlation with lactation from the 1st to 55th days in the term (*r* = 0.50; *p* < 0.05) group and did not show any correlation with lactation from the 2nd to 40th days in the preterm milk group (Figure 2C,D).

In contrast to milk IgG, 200 ng of lactating mothers’ plasma IgG was weakly recognized by SNA ((0.11 ± 0.01) AU) (Table 2).

### 3.3. RCA-Reactive Glycoform of Milk Igg

The mean value of relative RCA reactivity with IgG of the term milk samples ((1.65 ± 0.18) AU) (Figure 1C) was significantly higher than the values for the very ((1.46 ± 0.16) AU; *p* < 0.000001) and moderate ((1.45 ± 0.28) AU; *p* < 0.000001) preterm milk groups (Figure 1C).

The expression of RCA-reactive glycotopes on milk IgG did not show a correlation with lactation from the 1st/2nd to the 55th/40th days in the term (*r* = −0.2; *p* > 0.05) and preterm (*r* = 0.2; *p* > 0.05) groups (Figure 2E,F).

During all analyzed periods of lactation, the RCA reactivity with term milk IgG was significantly higher than preterm milk IgG. In the early colostrum groups, the relative reactivity of RCA with term and preterm IgG was 1.64 ± 0.24 and 1.41 ± 0.18 AU (*p* < 0.005), and remained almost at the same level in the colostrum samples at 1.67 ± 0.15 and 1.54 ± 0.23 AU (*p* < 0.04), respectively. In subsequent stages of lactation, the reactivity of RCA with IgG still remained at a similar level in the transitional ((1.63 ± 0.13 and 1.33 ± 0.43) AU, *p* < 0.00003, respectively) and mature ((1.61 ± 0.22 and 1.49 ± 0.17) AU, *p* < 0.005, respectively) term and preterm milk groups (Table 2).

In contrast to milk IgG, 200 ng of lactating mothers’ plasma IgG was very weakly recognized by RCA ((0.08 ± 0.03) AU) (Table 2).

### 3.4. GSL-Reactive Glycoform of Milk IgG

The relative reactivity of GSL with milk IgG (Figure 1D) of mothers having a newborn at term ((0.29 ± 0.21) AU) and for those of the very and moderate preterm milk groups were found to be nearly at the same level ((0.26 ± 0.11 and 0.28 ± 0.21) AU, respectively) (Figure 1D).

The expression of GSL-reactive glycotopes on IgG showed a weak positive correlation with lactation from the 1st to 55th days in the term (*r* = 0.3; *p* < 0.05) group and did not show a correlation with lactation from the 2nd to 40th days in the group preterm milk samples (Figure 2G,H).

In the early colostrum group of term and preterm milk samples, the relative reactivity of GSL with IgG was low ((0.23 ± 0.19 and 0.20 ± 0.15) AU, respectively), was significantly higher for term ((0.31 ± 0.25) AU; *p* < 0.03), and non-significantly higher for preterm ((0.39 ± 0.32) AU) colostrum groups. During further milk maturation stages, the GSL reactivity with IgG of the term and preterm transitional ((0.28 ± 0.15 and 0.29 ± 0.20) AU, respectively) and mature ((0.33 ± 0.22 and 0.24 ± 0.11) AU, respectively) milk samples remained at almost an unchanged level (Table 2).

In contrast to milk IgG, 200 ng of lactating mothers’ plasma IgG was almost unrecognized by GSL ((0.01) AU) (Table 2).

### 3.5. Sialylated and Asialylated Glycoforms of IgG in Preterm Milk in Relation to Infection of Mothers

**Figure 3:**
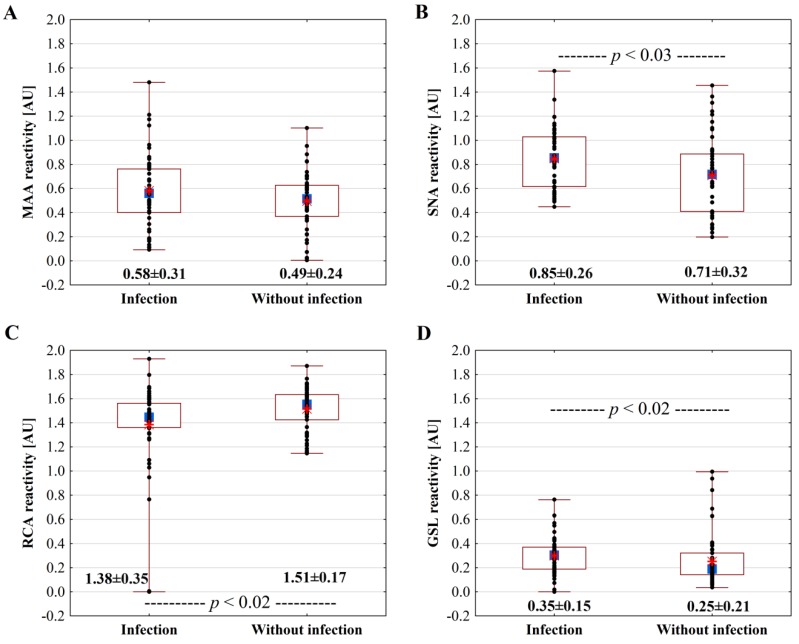
Relative amounts (AU) of MAA- (**A**), SNA- (**B**), RCA- (**C**), and GSL- (**D**) reactive IgG glycoform in skim preterm milk of mothers with and without infection. For experimental details see under Figure 1. Median (■), 25%–75% (□), Min–Max (I), Sample (●), Mean (❇).

The mean values of the relative reactivity of SNA and GSL with the IgG in preterm milk of mothers with infections (Figure 3B,D) were significantly higher ((0.85 ± 0.26) AU; *p* < 0.03 and (0.30 ± 0.15) AU; *p* < 0.02, respectively) than those observed for the preterm milk group without infection ((0.71 ± 0.32 and 0.25 ± 0.21) AU, respectively). Moreover, the comparison of the mean values of the relative reactivity of SNA with the IgG in preterm milk of mothers without infections, in contrast to the preterm milk of mothers with infections, revealed significant differences (*p* < 0.00006) with term milk group ((0.90 ± 0.23) AU). However, no significant differences were found for the mean values of the relative reactivity of GSL with the IgG of term milk (0.29 ± 0.21) AU and preterm milk of mothers with and without infections. In contrast, the mean value of the RCA relative reactivity with IgG of preterm milk of mothers with infection was significantly lower (*p*< 0.02) than the value found for the preterm milk group without infection ((1.38 ± 0.35 and 1.51 ± 0.17) AU, respectively) (Figure 3C); nevertheless for both analyzed groups, this was significant lower (*p* < 0.000001) than for the term milk group (1.65 ± 0.18) AU.

The mean value of the relative reactivity of MAA with the IgG of preterm milk of mothers having infections was 0.58 ± 0.31 AU and was higher, but not significantly, than the value found for the preterm milk group without infection ((0.49 ± 0.24) AU) (Figure 3A); nevertheless for both analyzed groups, this was significantly higher (*p* < 0.000001) than for the term milk group (0.27 ± 0.15 AU).

## 4. Discussion

To the best of our knowledge, despite the undisputed importance of glycosylation for the function of IgGs, a detailed study analyzing the human milk IgG sialylation pattern has not been attempted. Due to the very low concentration of milk IgG, the commonly used techniques including high-performance liquid chromatography (HPLC) and mass spectrometry (MS), require isolation and cannot be used with sufficient efficiency when limited volumes of human preterm and term colostrum are available. In such situations, the highly sensitive microtiter plate assay using biotinylated lectins is used as an alternative procedure [5,6,8] and the obtained results are in agreement with those obtained using mass spectra [1,3,45,46].

Based on lectin-IgG-ELISA, we demonstrated that human skim milk IgG was highly reactive, in contrast to the poorly reactive plasma IgG of lactating mothers with α2,3- and α2,6-sialic acid specific lectins, namely MAA and SNA, respectively (Table 2), as well as with galactose and *N*-acetylglucosamine specific RCA and GSL lectins, respectively.

Previous studies concerning the analysis of the IgG glycome using specific lectins [47] and antibodies [48] have shown that the recognition and binding are limited due to the location of the main *N*-glycans in the inner part of the Fc CH2 domains, in contrast to the Fab glycans, which are easily available for such interactions. Such a phenomenon was also reported by Zhang et al. [1], who demonstrated that the reaction with sialic acid and galactose-specific lectins was present only for the intact Fab fragment, but was absent for the Fc portion of IgG. Our previous analysis of milk IgG fucosylation [8] also suggests that Fc-related *N*-glycans are concealed for lectin interactions. Moreover, plasma IgG contains *N*-glycans in its Fc fragment and only 15%–25% contains additional *N*-glycans attached to the Fab fragment. Apart from the main type of sialic acid linkage, namely α2,6 reported for serum IgG *N*-glycans, the presence of small amounts of α2,3-sialic acid was reported on the Fab portion of serum IgG in healthy mothers up to one year after delivery [22]. In light of the above, we can speculate that the lectin-based sialylation profile of milk IgG observed by us is limited and in fact reflects the glycans of the Fab fragment, since the Fc fragment glycans are poorly sialylated and additionally hidden inside the protein backbone and inaccessible for recognition by lectins. Additionally, the differences in the sialylation of milk IgG may result from its dual origin, namely local synthesis by milk plasma cells inside the alveolar lumen of the mammary gland and transport of IgG from maternal blood via neonatal Fc receptors (FcRn) to the alveolar lumen [49]. The qualitative and quantitative differences in the glycosylation profile of IgG produced locally have been reported previously for amniotic IgG fucosylation and sialylation [42] and for milk IgG fucosylation [8].

Interestingly, unlike a stable level of IgG [8], the sialylation pattern of milk IgG was related to lactation stage and the week of delivery (Figure 1, Table 2). For early colostrum, regardless of the week of pregnancy ending, there were no differences in the expression of both α2,3- and α2,6-linked sialic acid for early term and early preterm colostrum; nevertheless, the number of samples of early colostrum in the group of mothers who gave birth prematurely was low (*n* = 7). Moreover, for both term and preterm milk groups, the expression of α2,3 sialic acid was the lowest at the beginning of lactation. However, in subsequent stages of lactation, namely colostrum, transitional, and mature milk, the sialylation profile was different for term and preterm milk samples. The α2,3- and α2,6-sialylation of term milk IgG increased significantly over milk maturation up to the 55th day. Nevertheless, for preterm milk IgG, the trend was weaker and only concerned α2,3-sialylation. Surprisingly, for galactosylation, no significant associations were demonstrated, although the agalactosylation pattern of term milk was weakly correlated with the milk maturation stages. The glycosylation changes of IgG are related to physiological maturation processes at various stages of development, which are regulated by steroid hormones such as during pregnancy [22] and puberty of children [50]. Moreover, for bovine milk IgG, a lactation stage-related decrease in the α2,6-sialylation level was reported, according to the lectin-based method, while α2,3-linked sialic acid was almost undetectable [2].

Milk maturation-related differences in sialylation profile have been previously reported for human milk oligosaccharides [51,52,53], the overall pool of skim milk glycoproteins [7], and for some glycoproteins [5,6], but the tendency of the observed changes varies depending on the glycoprotein being analyzed or the type of sialic acid linkage. The overall of α2,6-sialylation decreased with milk maturation for skim milk proteins [7], fibronectin [6], and AGP [5], in contrast to the α2,3-sialylation level, which remained almost unchanged over lactation.

There is limited understanding of the regulation of the glycosylation of glycoproteins synthesized locally in the mature and immature mammary gland, but levels of physiologically variable steroid hormones, associated with pregnancy and lactation, responsible for milk synthesis, may have an impact on the regulation of milk IgG glycosylation [54]. In light of the above, the alterations observed by us are not unexpected and have been reported previously for skim milk IgG fucosylation produced by the immature mammary gland [8]. Due to preterm birth, the mammary gland is not fully transformed to produce milk. The cellular and biochemical processes, which are responsible for preparing the gland for milk production and secretion, are regulated by a set of lactation-related hormones, namely estrogen, progesterone, and prolactin [55]. The alternation of the sialylation and asialylation profile of preterm milk IgG seems to be the consequence of different levels of estrogen, resulting in modulation of the glycosylation process, namely the expression and activity of enzymes. In the murine model, during lactation, ST6Gal induction takes place due to de novo recruitment of a normally silent promoter [56].

The degree of prematurity, namely very and moderately preterm, also has an impact on the sialylation profile of breast milk biomolecules. However, the observed changes were dependent on the type of sialic acid linkage (Figure 1). Alpha 2,3-sialylation was significantly elevated for both very and moderate preterm milk IgG, while alpha2,6-sialylation for moderate preterm milk was significantly lower and unchanged for very preterm milk in comparison to term milk. Similarly, the galactosylation pattern of preterm milk IgG was significantly lower in comparison to the term milk, regardless of the degree of prematurity.

Preterm birth is burdened with an increased risk of infection. In this context, the sialylation of IgG, which determines the functionality of molecules, having a steering role for pro- and anti-inflammatory IgG properties, should be taken into account. Although the concentration of milk IgG is relatively stable independently from the pathophysiological status of the lactating mother, the infection-related altered milk IgG profile was reported previously for the fucosylation of preterm milk IgG [8]. Moreover, IgG glycans located in the Fab region are important in the modulation of immunity, although the process is still poorly understood [23,57]. Using lectin-based analysis of the overall sialylation profile of milk IgG, the higher α2,6-sialylated and GSL-reactive (agalactosylated) and the lower of RCA-reactive (galactosylated) milk IgG glycotopes were found to be affected by the infection of lactating mothers who gave birth prematurely (Figure 3). However, the differences observed for inflammation-related α2,3-sialic acid were not statistically significant due to the large scatter of results for individual samples. Since the distinct patterns of IgG Fab glycosylation are associated with pathophysiological conditions (Table 2) (reviewed in [23]) as well as with pregnancy-associated hormonal changes [22], it is not surprising that the observed sialylation/asialylation pattern of the milk IgG of mothers with infection was altered. Moreover, acute systemic inflammation is associated with a very individualized galactosylation profile of plasma IgG glycans [58]. Such extensive changes observed in the milk of mothers with infection might highlight its importance in non-physiological conditions. However, the sialylation pattern is the result of the overlapping of various mechanisms (i.e. compensatory for the preterm and additionally inflammatory), but it seems that both remain under hormonal control.

One limitation of our study is the examination of glycotopes in the native molecules of milk IgG, which are not hidden inside the molecule and are available for recognition and binding by specific lectins used for determination. For this reason, the results obtained for the biomolecule in the native state may differ from the results obtained for the denatured form, which also provide information on glycotopes not available for reaction with lectins (i.e. hidden within the native structure). Moreover, the lectin-based results are expressed as the relative reactivity, since adequate standards are not available.

A strength of our study is the large and unique collection of milk samples of mothers who delivered prematurely and at term including all stages of milk maturation (i.e. from early colostrum to fully mature milk), which has an impact on credibility of the results obtained. Moreover, using sialic acid-specific lectins such as MAA and SNA allows for the determination of the α2,3- and α2,6-sialylation profile of milk IgG available glycans, in contrast to expensive and time- and sample-consuming instrumental methods. Such an approach is very useful to compare the sialylation profile at different stages of lactation and to determine the possible impact of perinatal risk factors on sialylation pattern. Moreover, lectins seem to be a molecular probe used in molecular biology or chemistry to study the overall glycosylation pattern as well as the glycan structure of native glycoproteins.

## 5. Conclusions

Given the importance of bioactive glycoproteins for optimal immunological protection of breastfed newborns, further studies are required to find out what specific factors control the glycosylation process and are responsible for the observed variations. However, the method used should be chosen very carefully and should take into account the native state of the molecule that in fact participates in biological processes, and the available sugar part allows it to be recognized by lectin receptors actively participating in providing adequate immune protection. Lectin-based analysis reveals fundamental differences in the sialylation pattern of human milk IgG that have not been previously reported. The sialylation pattern of milk IgG is extremely important in light of the fact that the presence or absence of sialic acid is a key molecule that causes opposite functionality of IgG such as pro- and anti-inflammatory, respectively, and might be crucial for maturation of the newborn’s immature immune system. Moreover, we can speculate that milk IgG, due to the highly sialylated IgG glycotopes, similar to S-IgA glycotopes, might provide IgG with supplementary bacterial lectin-binding sites apart from binding sites in the Fab fragment, and in that way enable IgG to participate in both innate and adaptive immunity.

## Figures and Tables

**Table 1:** Major binding specificities of used lectins.

Origin and Abbreviation of Used Agglutinin	Binding Preference	References
*Maackia amurensis* II (MAA II)	Neu5Ac(α2,3)Gal(β1,4)GalNAc	[36,37]
*Sambucus nigra* (SNA)	Neu5Ac(α2,6)Gal/GalNAc	[1,37,38]
*Ricinus communis* I (120) (RCA I (120))	Gal(β1,4)GlcNAc no affinity for agalactosylated *N*-type	[1,37,39]
*Griffonia simplicifolia* II (GSL II)	GlcNAc (agalactosylated glycans) no affinity for fully galactosylated	[1,37,40]

**Table 2:** Relative amounts of sialyl-IgG glycoforms during lactation in milk from mothers giving birth to term and preterm infants.

**No**		**IgG Reactivity [AU] with Lectins**			
		MAA	SNA	RCA	GSL
		specific to a monosaccharide unit linked by glycosidic linkages			
		Neu5Ac(α2,3) Gal(β1,4)GalNAc	Neu5Ac(α2,6) Gal/GalNAc	Gal	GlcNAc
1. Early colostrum days 1–3	Term	0.16 ± 0.12 0.12 (0.08–0.26) *n* = 35	0.75 ± 0.17 0.74 (0.63–0.84) *n* = 35	1.64 ± 0.24 1.70 (1.57–1.81) *n* = 39	0.23 ± 0.19 0.17 (0.11–0.24) *n* = 39
	Preterm	0.09 ± 0.08 0.09 (0.02–0.16) *n* = 7	0.84 ± 0.19) 0.87 (0.62–1.02) *n* = 7	1.41 ± 0.18 1.45 (1.21–1.52) *n* = 7 *p* < 0.005^a^	0.20 ± 0.15 0.16 (0.04–0.38) *n* = 7
2. Colostrum days 4–7	Term	0.27 ± 0.15 0.24 (0.15–0.38) *n* = 65 *p* < 0.0004^1T^	0.89 ± 0.20 0.88 (0.75–0.99) *n* = 65 *p* < 0.0005^1T^	1.67 ± 0.15 1.68 (1.58–1.76) *n* = 75	0.31 ± 0.25 0.29 (0.14–0.43) *n* = 74 *p* < 0.03^1T^
	Preterm	0.41 ± 0.35 0.34 (0.18–0.52) *n* = 14 *p* < 0.005^1P^	0.73 ± 0.30 0.65 (0.55–0.84) *n* = 14 *p* < 0.03^b^	1.54 ± 0.23 1.61 (1.40–1.68) *n* = 15 *p* < 0.04^b^	0.39 ± 0.32 0.34 (0.08–0.69) *n* = 15
3. Transitional milk days 8–14	Term	0.28 ± 0.13 0.25 (0.20–0.36) *n* = 40	1.00 ± 0.25 1.00 (0.91–1.14) *n* = 40 *p* < 0.006^2T^	1.63 ± 0.13 1.64 (1.55–1.70) *n* = 43	0.28 ± 0.15 0.24 (0.17–0.37) *n* = 43
	Preterm	0.63 ± 0.24 0.54 (0.46–0.71) *n* = 26 *p* < 0.003^2P^ *p* < 0.000001^c^	0.81 ± 0.36 0.83 (0.58–0.98) *n* = 26 *p* < 0.008^c^	1.33 ± 0.43 1.42 (1.30–1.58) *n* = 28 *p* < 0.00003^c^	0.29 ± 0.20 0.22 (0.16–0.38) *n* = 28
4. Mature milk days 15–55/40	Term	0.38 ± 0.13 0.40 (0.27–0.45) *n* = 28 *p* < 0.004^3T^	0.97 ± 0.27 1.04 (0.76–1.17) *n* = 28	1.61 ± 0.22 1.64 (1.49–1.79) *n* = 29	0.33 ± 0.22 0.27 (0.13–0.50) *n* = 29
	Preterm	0.58 ± 0.22 0.59 (0.47–0.74) *n* = 47 *p* < 0.00002^d^	0.76 ± 0.28 0.77 (0.51–1.01) *n* = 47 *p* < 0.004^d^	1.49 ± 0.17 1.51 (1.43–1.60) *n* = 55 *p* < 0.005^d^	0.24 ± 0.11 0.21 (0.16–0.32) *n* = 55
5. Lactating mother’s plasma		0.00 ± 0.00 *n* = 40	0.11 ± 0.01 0.11 (0.11–0.12) *n* = 40	0.08 ± 0.03 0.075 (0.057–0.1) *n* = 40	0.01 ± 0.0 0.04 *n* = 40

The reactivity of 200 ng of human milk or lactating mother’s plasma IgG with *Maackia amurensis* agglutinin (MAA) (specific to α2,3-linked sialic acid), *Sambucus nigra* agglutinin (SNA) (specific to α2,6-linked sialic acid), *Ricinus communis* agglutinin I (RCA I) (specific to β1,4-linked galactose), and *Griffonia simplicifolia* agglutinin II (GSL II) (specific to *N*-acetylglucosamine) is expressed as the absolute value of absorbance units (AU) at 405 nm based on the lectin–IgG-ELISA as described in the Materials and Methods. *n* – number of samples. Values are given as the mean ± SD and median and 25th–75th percentiles in parentheses. The Mann–Whitney U test was used for statistical calculations, and a *p*-value lower than 0.05 was regarded as significant. The numbers of samples differed between one lectin and one another due to the limited volume of milk samples used for analysis. Significantly different from the milk sample group of: ^1T^ term early colostrum (1–3 days), ^2T^ term colostrum (4–7 days), ^3T^ term transitional milk, ^1P^ preterm early colostrum (2–3 days), ^2P^ preterm colostrum,^a^ term early colostrum, ^b^ term colostrum, ^c^ term transitional milk, ^d^ term mature milk.
